# Fucoxanthin Exerts Anti-Tumor Activity on Canine Mammary Tumor Cells via Tumor Cell Apoptosis Induction and Angiogenesis Inhibition

**DOI:** 10.3390/ani11061512

**Published:** 2021-05-23

**Authors:** Hyuk Jang, Jawun Choi, Jeong-Ki Park, Gayeon Won, Jae-Won Seol

**Affiliations:** College of Veterinary Medicine, Jeonbuk National University, Iksan 54596, Jeollabuk-do, Korea; hjang1003@naver.com (H.J.); jwchoi529@naver.com (J.C.); bjk819@naver.com (J.-K.P.)

**Keywords:** fucoxanthin, canine mammary gland tumor, angiogenesis, angiopoietin-2, apoptosis

## Abstract

**Simple Summary:**

Fucoxanthin is a carotenoid that reportedly exhibits anticancer activity against different types of cancer cells. However, the activity of fucoxanthin in canine mammary gland tumors has not been extensively investigated. In this study, we evaluated fucoxanthin against canine mammary tumor cells (CMT-U27) and human umbilical vein endothelial cells (HUVECs). Our results indicated that fucoxanthin induced apoptosis via caspase activation and suppressed angiogenesis in CMT-U27 cells. Moreover, fucoxanthin inhibited tube formation and cell migration in HUVECs and CMT-U27 cells, indicating that it possessed anti-angiogenic potential. In conclusion, fucoxanthin induced tumor cell death and inhibited angiogenesis. Therefore, we propose that fucoxanthin can be considered a prospective therapeutic agent for canine mammary gland tumors.

**Abstract:**

Fucoxanthin is a carotenoid derived from brown algae. It is known to exhibit anticancer activity, including the promotion of apoptosis and cell cycle arrest in several tumors. However, it remains unclear whether fucoxanthin exhibits anticancer activity against mammary gland tumors. In this study, we evaluated fucoxanthin activity against canine mammary tumor cells (CMT-U27) and human umbilical vein endothelial cells (HUVECs) to investigate its effect on cell viability, migration, tube formation, and angiopoietin 2 (Ang2) expression. Our results showed that fucoxanthin induced apoptosis via caspase activation in CMT-U27 cells. In rat aortic ring assay, fucoxanthin suppressed endothelial cell sprouting. Furthermore, fucoxanthin inhibited tube formation and migration in HUVECs. The number of migrated cells was assessed using CMT-U27 cells. The results demonstrated that fucoxanthin exerted anti-angiogenic activity on HUVECs and CMT-U27 cells by promoting Ang2 expression. In conclusion, our results demonstrated that fucoxanthin induced tumor cell death and inhibited angiogenesis, suggesting that fucoxanthin could be considered as a promising therapeutic agent for canine mammary gland tumors.

## 1. Introduction

Canine mammary gland tumors are commonly reported in female dogs, and approximately 50% of these tumors are malignant [1,2]. They occur more frequently in non-neutered female dogs of 10–11 years of age [3]. They are often caused by changes in estrogen receptors and are primarily excised surgically [4]. Piroxicam and deracoxib are currently being investigated as prospective chemotherapeutic agents for these tumor types [5,6]; however, their clinical application remains debatable. Additionally, several side effects associated with these chemotherapeutic agents have been reported [7]. Therefore, the identification of natural product-derived drugs exhibiting safety and easy consumption features is crucial.

Fucoxanthin is the main carotenoid extracted from brown algae [8]. It possesses several biological activities, such as anti-inflammatory, antioxidant, antidiabetic, hepatoprotective, and anti-tumor activities [9]. Fucoxanthin induces apoptosis and cell cycle arrest in various cancer cells, including human colon cancer (HCT116), human hepatocellular carcinoma (HepG2), human prostate cancer (LnCaP and DU145), and human promyelocytic leukemia (HL-60) cells [10,11,12,13]. Furthermore, it also reduces the protein expression of Bcl-2, an anti-apoptotic protein, in prostate cancer and human promyelocytic leukemia [11,14,15]. Moreover, the anti-angiogenic activity of several natural products has been reported in various cancer cells [16,17,18]. However, no evidence exists regarding the anti-tumor potential of fucoxanthin in canine mammary gland tumors.

Angiogenesis refers to the process by which new blood vessels are formed from existing blood vessels [19]. It plays an important role in the progression of diseases, such as cancer and rheumatoid arthritis [20,21], as well as in the wound healing process [22,23]. Research is being actively conducted on angiogenesis inhibition, because it is known to be an essential process in tumor growth and metastasis [24].

In this study, we aimed to examine the potential of fucoxanthin in the promotion of cell apoptosis and inhibition of angiogenesis in canine mammary tumor cells (CMT-U27) and human umbilical vein endothelial cells (HUVECs). Our results suggest that fucoxanthin is a promising agent with applicability in the effective and safe treatment of canine mammary gland tumors.

## 2. Materials and Methods

### 2.1. Cell Cultures and Reagents

CMT-U27 cells were purchased from American Type Culture Collection (ATCC) (Manassas, VA, USA) and cultured in RPMI 1640 medium (Gibco, Grand Island, NY, USA) supplemented with 10% fetal bovine serum (Atlas Biologicals, For Collins, CO, USA). Penicillin (100 U/mL) and streptomycin (100 μg) were purchased from Sigma-Aldrich (St. Louis, MO, USA). HUVECs and their growth medium (EGM-2 MV BulletKit^TM^) were purchased from Lonza Group Ltd. (Basel, Switzerland). HUVECs were used in the passage numbers 4–5 in all experiments. All cells were incubated at 37 °C in 5% CO_2_. Fucoxanthin and dimethyl sulfoxide was purchased from Sigma-Aldrich (St. Louis, MO, USA). Fucoxanthin was dissolved in dimethyl sulfoxide to obtain a 20 mM stock solution.

### 2.2. Cell Viability Assay

CMT-U27 cells were cultured in 6-well plates at a density of 5 × 10^5^ cells and incubated at 37 °C for 24 h. Seeded cells were treated with 0, 5, 10, and 20 μM of fucoxanthin and incubated for 24 h. Cell morphology was observed under an inverted microscope (Nikon Corporation, Tokyo, Japan). Crystal violet staining was performed to assess cell viability. In this method, viable cells were subjected to staining procedures using a crystal violet staining solution (0.5% crystal violet in 30% ethanol and 3% formaldehyde) for 10–15 min at room temperature (RT); thereafter, the cells were subjected to washing steps 3–4 times using distilled water and were subsequently subjected to drying. The incorporated dye was solubilized using 1% sodium dodecyl sulfate. The absorbance was measured at 550 nm using a spectrophotometer (Bio-Rad Laboratories, Inc., Hercules, CA, USA).

### 2.3. Annexin V Assay

Cell apoptosis was assessed by conducting a flow cytometric annexin V assay (Santa Cruz Biotechnology, Inc., Dallas, TX, USA) according to the manufacturer’s instructions. The annexin V content was determined by measuring fluorescence at 488 nm (excitation) and 525 nm (emission) using the Guava easyCyte HT system (Millipore, Billerica, MA, USA).

### 2.4. Western Blotting

At the indicated time, CMT-U27 cells were subjected to homogenization using chilled lysis buffer containing a protease inhibitor cocktail (Sigma-Aldrich). Proteins were subjected to separation using SDS-PAGE and transferred onto nitrocellulose membranes. After conducting blocking using 5% skim milk, the membranes were incubated overnight at 4 °C with the following primary antibodies in the blocking buffer: anti-poly (ADP-ribose) polymerase (PARP) (rabbit), anti-caspase 8 (mouse; both from Cell Signaling Technology, Inc., Beverly, MA, USA), and anti-β-actin (mouse monoclonal; Sigma-Aldrich). Membranes were then incubated with Horseradish Peroxidase (HRP)-conjugated secondary antibodies for 1 h at RT. Chemiluminescent signals were developed using the HRP substrate (Millipore) and detected using the Fusion FX7 acquisition system (Vilbert Lourmat, Eberhardzell, Germany).

### 2.5. Rat Aortic Ring Assay

The rat aortic ring assay was performed following a previously published protocol with slight modifications to confirm the effects of fucoxanthin in the ex vivo angiogenesis study [25]. Thoracic aortas were removed from sacrificed Sprague Dawley rats (4-week-old, Samtako) under sterile conditions, and rinsed using cold PBS. Aortic rings (1 mm long) were sectioned with a surgical blade. Each ring was implanted on a Matrigel-pre-coated 24-well-plate. The complete Dulbecco’s modified Eagle’s medium was added to the wells in an absence or presence of fucoxanthin. Seven days after the treatment, the rings were observed under a microscope (Nikon Corporation), and then microvessel sprouting was estimated. The experimental protocol for this study was approved by the Institutional Animal Care and Use Committee (IACUC) of Jeonbuk National University (Approval number. 2021-074).

### 2.6. In Vitro Tube Formation Assay

Matrigel^TM^ (Corning Inc., New York, NY, USA) was subjected to thawing overnight at 4 °C for the tube formation assay. Matrigel solidification on a 24-well plate was performed at 37 °C for 30 min. HUVECs were seeded at a density of 1 × 10^5^ cells/well in a growth medium in an absence or presence of fucoxanthin. The cells were then incubated at 37 °C for an additional period of 18 h. Tube formation on the Matrigel was observed under a microscope (Nikon Corporation), pictured, and quantified by manually counting the number of tubules from three different photographs for each condition.

### 2.7. In Vitro Migration Assay

HUVECs and CMT-U27 cells were seeded in 6-well plates. After reached 100% confluence, cells were wounded manually using a sterile 1000 μL tip and washed using sterile PBS. The fresh medium containing 1% fetal bovine serum was replaced with different concentrations of fucoxanthin. After the indicated time, the wound closure by cell migration was photographed using a microscope (Nikon Eclipse TS100; Nikon Corporation).

### 2.8. Reverse Transcription Polymerase Chain Reaction (RT-PCR)

RNA extraction (2 µg) from each sample were reverse-transcribed into cDNA according to the manufacturer’s instructions (Cat. 18080093, Invitrogen). The cDNA aliquots were amplified using Go Taq DNA polymerase (Promega) in the Mycyler Thermal Cycler (Bio-Rad); the primers are listed in Table 1. The thermocycling conditions of the RT-PCR cycle were set to the following: 94 °C for 1 min, 60 °C for 1 min, and 72 °C for 1 min. Equal volumes of the products containing the LoadingSTAR nucleic acid dye (6X, Dynebio, Seongnam, Korea) were loaded onto 1.5% agarose gel and conducted electrophoresis. The gel was subjected to electrophoresis and then imaged using the Fusion FX7 acquisition system (Vilbert Lourmat).

### 2.9. Immunocytochemistry

CMT-U27 cells and HUVECs were seeded on coverslips coated with 1% gelatin. The cells were fixed by 2% paraformaldehyde, permeabilized by 0.5% Triton X-100 in PBS for 5 min, and blocked in 5% animal serum (donkey) in PBS containing 1% bovine serum albumin for 1 h at RT. The cells were incubated overnight at 4 °C with anti-VE-cadherin (goat; R&D Systems) and anti-angiopoietin 2 (Ang2) (rabbit polyclonal; Proteintech) antibodies. Cells were incubated with FITC-conjugated donkey anti-goat IgG and Cy3-conjugated donkey anti-rabbit IgG (both from Jackson ImmunoResearch). Nuclei were subjected to staining using 4′,6-diamidino-2-phenylindole. Then, the cells were mounted with the mounting medium (Dako, Carpinteria, CA, USA), and immunofluorescence images were taken by a confocal microscope (Carl Zeiss, Inc., Jena, Germany).

### 2.10. Statistical Analyses

The mean values of all experiments are represented with the standard deviation. For multigroup analysis of variance, one-way ANOVA followed by Bonferroni post hoc test was conducted. Statistical significance was set as less than 0.05 and is represented as * *p* < 0.05, ** *p* < 0.01, and *** *p* < 0.001.

## 3. Results

### 3.1. Fucoxanthin Reduces Cell Viability in CMT-U27 Cells

**Figure 1:**
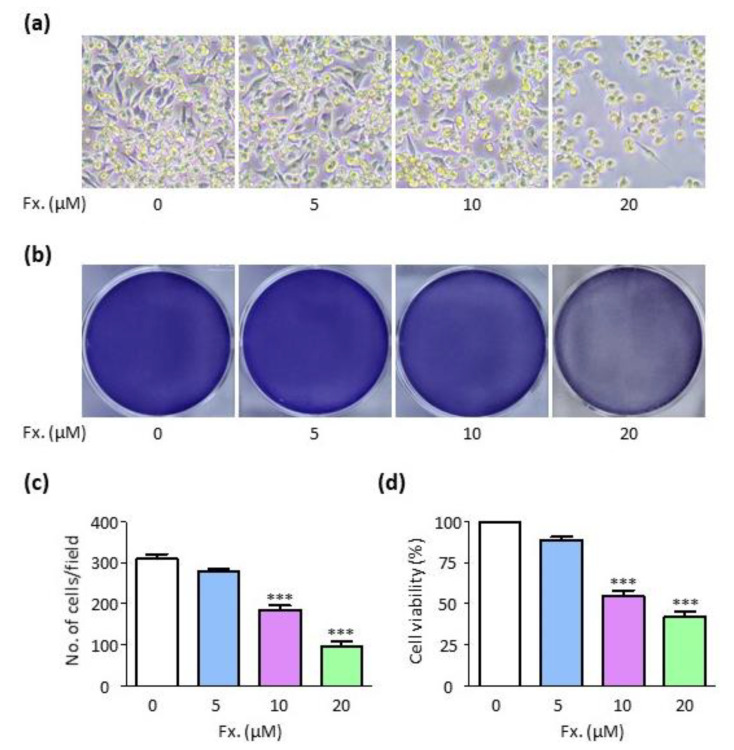
Fucoxanthin inhibits cell viability in CMT-U27 cells. (**a**,**b**) Images showing morphological changes (**a**) and crystal violet-stained cells (**b**) on CMT-U27 cells after fucoxanthin-treatment (0, 5, 10, and 20 μM) for 24 h. Magnification, 100×. (**c**) Number of viable cells per field from (**a**). (**d**) Quantification of CMT-U27 cells’ viability from (**b**). Fx., Fucoxanthin. Values are presented as the mean ± SD of three independent experiments. *** *p* < 0.001.

To investigate the activity of fucoxanthin in tumor cells, a cell viability assay was performed. CMT-U27 cells were treated with different concentrations (0, 5, 10, and 20 μM) of fucoxanthin for 24 h. It was observed that cell viability decreased in a concentration-dependent manner (Figure 1a, b), which was indicated by the decrease in the number of viable cells per unit area (Figure 1c). These results showed that fucoxanthin suppressed the viability of CMT-U27 cells in a concentration-dependent manner.

### 3.2. Fucoxanthin Causes Tumor Cell Death by Inducing Apoptosis

**Figure 2:**
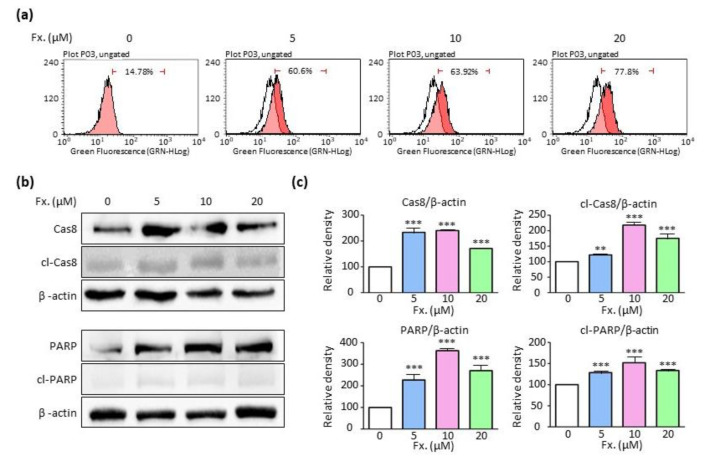
Fucoxanthin induces cell apoptosis via the caspase-dependent pathway. (**a**) Flow cytometry analysis showing apoptosis in CMT-U27 cells treated with fucoxanthin (0, 5, 10, and 20 μM) for 24 h. (**b**) Western blot images showing protein expression of caspase 8, cleaved-caspase 8, PARP, and cleaved-PARP in CMT-U27 cells after fucoxanthin treatment (0, 5, 10, and 20 μM) for 24 h. (**c**) Relative density of PARP, cleaved-PARP, caspase 8, and cleaved-caspase 8 normalized by β-actin. Fx., Fucoxanthin. Values are presented as the mean ± SD of three independent experiments. ** *p* < 0.01, *** *p* < 0.001.

Flow cytometry and Western blotting experiments were performed to investigate whether fucoxanthin induced apoptosis. Flow cytometry results indicated that the abundance of annexin V-positive cells was five times higher in cells treated with 20 μM fucoxanthin when compared to untreated cells (Figure 2a). Fucoxanthin also enhanced the relative density of apoptosis-related proteins such as caspase 8, cleaved-caspase 8, PARP, and cleaved-PARP (Figure 2b,c and Figure S1). These results revealed that fucoxanthin induced apoptosis in CMT-U27 cells in a concentration-dependent manner.

### 3.3. Fucoxanthin Suppresses Endothelial Cell Sprouting and Tube Formation

**Figure 3:**
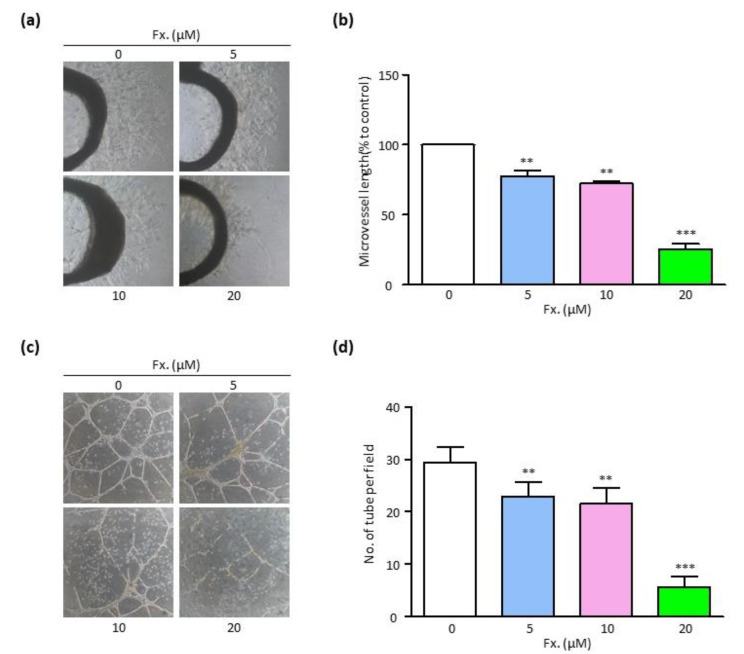
Fucoxanthin inhibits endothelial cell sprouting and tube formation. (**a**,**b**) Images showing sprouting of microvessels from rat aorta (**a**) and comparison of maximal length of microvessel (**b**) in treatment with fucoxanthin (0, 5, 10, and 20 μM) for 7 days. Magnification, 40×. (**c**,**d**) Images showing EC tube formation (**c**) and comparison of the number of tubules (**d**) in treatments with fucoxanthin (0, 5, 10, and 20 μM) for 18 h. Magnification, 100×. Fx., Fucoxanthin. Values are presented as the mean ± SD of three independent experiments. ** *p* < 0.01, *** *p* < 0.001.

To confirm the anti-angiogenic activity of fucoxanthin, the rat aortic ring and tube formation assays were performed. The rat aortic ring assay was performed to determine the ex vivo activity of fucoxanthin on microvessel sprouting. It was confirmed that fucoxanthin reduced microvascular sprouting by 25% when compared to untreated cells (Figure 3a,b). Furthermore, the tube formation assay showed that fucoxanthin significantly inhibited tubule formation in HUVECs (Figure 3c,d). These results indicated that fucoxanthin exhibited anti-angiogenic activity, which prevented the sprouting of new blood vessels.

### 3.4. Fucoxanthin Inhibits the Cell Migration in CMT-U27 Cells and HUVECs

**Figure 4:**
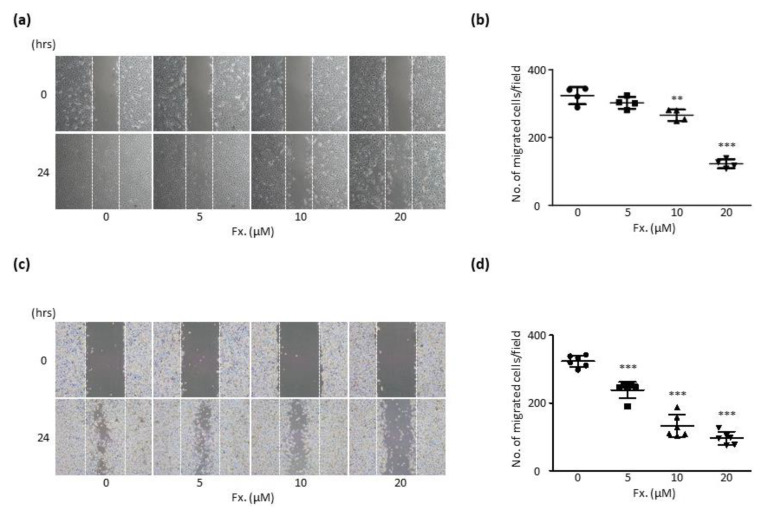
Fucoxanthin decreases the cell migration in HUVECs and CMT-U27 cells. (**a**,**c**) Images showing HUVEC (**a**) and CMT-U27 cell (**c**) migration after fucoxanthin (0, 5, 10, and 20 μM) for 24 h. Magnification, 40×. (**b**,**d**) Quantification of HUVEC (**b**) and CMT-U27 cell (**d**) migration from (**a**,**c**), respectively. Fx., Fucoxanthin. Values are presented as the mean ± SD of three independent experiments. ** *p* < 0.01, *** *p* < 0.001.

To confirm the anti-metastatic activity of fucoxanthin in a comprehensive manner, a migration assay was performed using CMT-U27 cells and HUVECs. The migration assay results showed that fucoxanthin inhibited the migration of cells in a time-dependent and concentration-dependent manner. In both cells that were not subjected to treatments with fucoxanthin, migration actively occurred even after 24 h, up to a point when the scratched area showed overpopulation of the cells. After subjection to fucoxanthin treatment for 24 h, the number of migrated cells was markedly lower when compared to untreated cells. In cells treated with 20 μM fucoxanthin for 24 h, the number of migrated cells reduced by 37.92% in HUVECs and by 29.66% in CMT-U27 cells when compared to untreated cells (Figure 4a–d). These results indicate that fucoxanthin exerted an anti-angiogenic activity by inhibiting cell migration.

### 3.5. Fucoxanthin Regulates Ang2 Expression in the Absence of the Vascular Endothelial Growth Factor A (VEGF-A)/Vascular Endothelial Growth Factor Receptor 2 (VEGFR-2) Signaling Pathway

**Figure 5:**
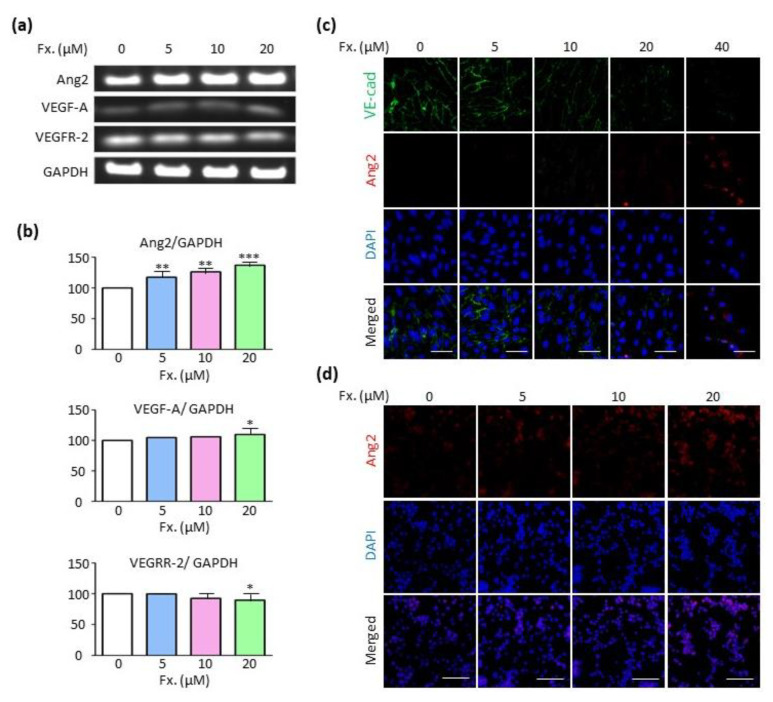
Fucoxanthin regulates Ang2 expression in the absence of VEGF-A/VEGFR-2 signaling pathway. (**a**) Images showing Ang2, VEGF-A, and VEGFR-2 mRNA expression in HUVECs after fucoxanthin treatment (0, 5, 10, and 20 μM) for 24 h. (**b**) Relative density of Ang2, VEGF-A, VEFR-2 normalized by GAPDH. (**c**,**d**) Immunocytochemistry images showing Ang2 protein expression in HUVECs (**c**) and CMT-U27 cells (**d**) after fucoxanthin treatment at the indicated concentration for 24 h. Fx., Fucoxanthin. Values are presented as the mean ± SD of three independent experiments. * *p* < 0.05, ** *p* < 0.01, *** *p* < 0.001.

To decipher the anti-angiogenic mechanism of fucoxanthin, the mRNA levels of Ang2, VEGF-A, and VEGFR-2, which are factors related to angiogenesis, were determined. It was observed that fucoxanthin increased Ang2 levels, whereas the levels of VEGF-A and VEGFR-2 were almost unaltered (Figure 5a,b). Immunocytochemistry was performed for visualizing the expression of Ang2 in cells. The Ang2 expression significantly increased in HUVECs treated with 40 μM fucoxanthin (Figure 5c) and in CMT-U27 cells treated with 20 μM fucoxanthin (Figure 5d). Furthermore, VE-cadherin is a component of endothelial cell-to-cell adherence junction, and it has a key role in the maintenance of vascular integrity [26]. To confirm the effects of fucoxanthin on cell adhesion, we checked VE-cadherin expression. As a result, fucoxanthin reduced the protein expression of VE-cadherin in a dose-dependent manner, showing that fucoxanthin weakened the cell-to-cell junction. Thus, an increase in Ang2 expression may be the probable mechanism of anti-angiogenic actions of fucoxanthin.

## 4. Discussion

Canine mammary gland tumors pose considerable health concerns, because approximately 50% of these tumor types transform into malignant entities. They are often caused by changes in the estrogen receptors and occurrences are commonly reported in non-neutered female dogs. Although Non-steroidal anti-inflammatory drugs (NSAIDs) are used for treating these tumor types, several side effects have been reported [27]. Therefore, the use of natural products for the treatment of these tumor types has recently attracted attention. Particularly, previous studies have shown that natural products exhibit anti-angiogenic activity, and this activity can lead to the exertion of anti-tumor activity [28,29,30]. Therefore, we studied the activity of fucoxanthin, a natural product, on angiogenesis and tumor cell death in CMT-U27 cells and HUVECs.

Fucoxanthin reportedly demonstrates apoptotic activity in various cell types. As shown in Figure 1, cell viability was significantly reduced when cells were treated with 10 μM and 20 μM fucoxanthin for 24 h. Additionally, the number of viable cells per unit area was also reduced. These results showed that fucoxanthin caused cell death in CMT-U27 cells. Cell death is largely divided into three main types, namely, apoptosis, autophagy, and necrosis [31,32]. Flow cytometry was performed to determine the types of cell death that occurred. It was observed that the number of annexin V-positive cells significantly increased in a concentration-dependent manner, indicating that fucoxanthin induced apoptosis. Apoptosis is accurately regulated by many protein molecules, including caspase 3, caspase 7, caspase 8 and PARP. Among them, caspase-8 plays the role of apoptosis promoter, especially in an extrinsic way. Caspase-8 is coupled with FADD, and then activates a cascade. On the other hand, PAPR is a family of proteins involved in many cellular processes such as DNA repair, genomic stability, and programmed cell death. PARP cleavage plays a positive regulatory role in apoptosis and is considered one of the biomarkers for the detection of apoptosis. In this study, the expression of apoptotic proteins, i.e., PARP, cleaved-PARP, caspase 8, and cleaved-caspase 8, was also elevated. Therefore, fucoxanthin induced cell death in CMT-U27 cells via apoptosis, which validated its anti-tumor potential.

Angiogenesis refers to the process of formation of new blood vessels sprouting from the existing blood vessels. Continuous creation of new blood vessels to maintain a specific microenvironment is imperative for the survival of tumors [33]; the angiogenesis process is essential for their existence. In other words, the suppression of angiogenesis is expected to result in anti-tumor activity. It was observed in the rat aortic ring assay that treatment with fucoxanthin inhibited the sprouting of new blood vessels, which was particularly prominent at a 20 μM concentration. As shown in Figure 3, the number of tubes per area decreased as the concentration of fucoxanthin increased. These results further confirmed the anti-angiogenic activity of fucoxanthin.

Cancer cells first proliferate sufficiently at the site of origin and then metastasize to lymphatic vessels and blood vessels through invasion and migration [34]. Therefore, migration is strongly associated with metastasis [35]; hence, anti-tumor activity may be expected by suppressing migration. In the migration assays conducted using HUVECs and CMT-U27 cells, fucoxanthin inhibited migration in both cells. These results demonstrated that fucoxanthin exhibited anti-migrative potential, suggesting its possible utility as an effective tumor metastasis inhibitor.

Factors related to angiogenesis include VEGF, epidermal growth factor, insulin-like growth factor, and Ang2 [36,37,38,39]. Ang2 plays different roles depending on the presence or absence of VEGF. In the presence of VEGF, Ang2 induces migration, proliferation, and sprouting, and inhibits endothelial cell death and vessel regression. In the absence of VEGF, Ang2 exerts the opposite effect [40]. Our results showed almost negligible levels of VEGF-A, whereas Ang2 levels increased, and VEGFR-2 levels showed less remarkable changes in HUVECs. Immunocytochemistry analysis revealed that high concentrations of fucoxanthin markedly increased Ang2 protein expression in HUVECs and CMT-U27 cells. Therefore, it may be proposed that an increase in Ang2 promotes the death of endothelial cells and inhibits the migration, proliferation, and sprouting of new blood vessels. It is also supported that fucoxanthin suppresses the expression of VE-cadherin, a junction protein between endothelial cells. Further studies are warranted for improved comprehension of the anti-angiogenic activity of fucoxanthin in canine vascular endothelial cells.

In conclusion, fucoxanthin demonstrates anti-angiogenic activity by inhibiting proliferation, migration, and tube formation in CMT-U27 cells and HUVECs. Furthermore, it can exhibit anti-tumor activity by increasing apoptosis in CMT-U27 cells. These results suggest that fucoxanthin can be a prospective therapeutic agent for canine mammary gland tumors.

## Figures and Tables

**Table 1:** Primer sequence used for RT-PCR.

Gene	Primer Sequence	Size (bp)
Ang2	5′-GGATCTGGGGAGAGAGGAAC-3′ 5′-CTCTGCACCGAGTCATCGTA-3′	535
VEGF-A	5′- TGCAGATTATGCGGATCAAACC -3′ 5′- TGCATTCACATTTGTTGTGCTGTAG -3′	81
VEGFR-2	5′-CCAGCAAAAGCAGGGAGTCTGT-3′ 5′-TGTCTGTGTCATCGGAGTGATATCC-3′	87
GAPDH	5′-ACCACAGTCCATGCCATCAC-3′ 5′-TCCACCACCCTGTTGCTGTA-3′	452

## Supplementary Materials

The following are available online at https://www.mdpi.com/article/10.3390/ani11061512/s1, Figure S1: Immunoblots and densitometry reading/intensity ratio of apoptotic markers in CMT-U27 cells.
