# Supplementary material for: Fucoxanthin Exerts Anti-Tumor Activity on Canine Mammary Tumor Cells via Tumor Cell Apoptosis Induction and Angiogenesis Inhibition
Source: Animals (Basel). 2021 May 23;11(6):1512. doi: 10.3390/ani11061512 (PMC8224559; doi:10.3390/ani11061512)
Supplement: Supplementary file 1 [file animals-11-01512-s001.zip › animals-1202644-supplementary.pdf]

animals

MDPI

a

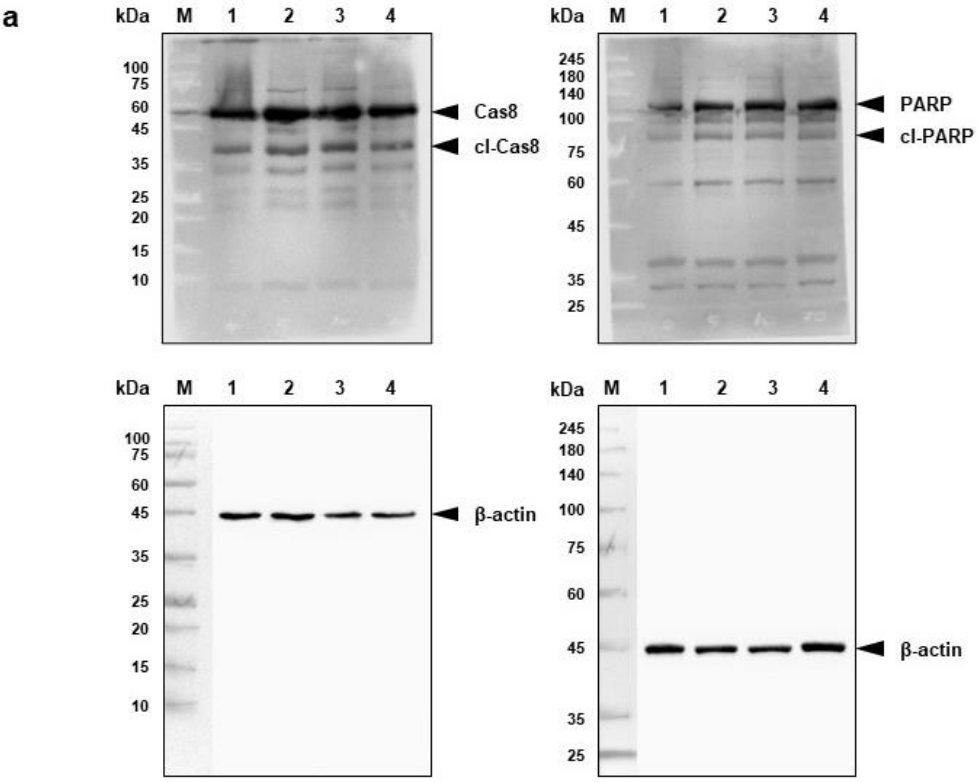

Lane M: Molecular weight markers
Lane 1: Untreated
Lane 2: Fucoxanthin (5 μg/ml)
Lane 3: Fucoxanthin (10 μg/ml)
Lane 4: Fucoxanthin (20 μg/ml)

b

CMT-U27				
**Fucoxanthin (μg/ml)**	**Cas8/β-actin**	**cl-Cas8/β-actin**	**PARP/β-actin**	**cl-PARP/β-actin**
**0**	1.323564002	0.527500311	1.350730415	0.576089245
**5**	3.224276246	0.633331105	2.979020537	0.738223759
**10**	3.296589588	1.121295213	4.866831468	0.890435399
**20**	2.335456798	0.924754964	3.513100665	0.804857263

Figure S1. Immunoblots and densitometry reading/intensity ratio of apoptotic markers in CMT-U27 cells. CMT-U27 cells were treated with 0, 5, 10, and 20 μg/ml of fucoxanthin for 24 h. The protein expression of cas8 (55 kDa), cleaved-cas8 (43 kDa), PARP (116 kDa), and cleaved-PARP (89 kDa) was analyzed by (**a**) western blots and (**b**) densitometry reading/intesntiy ratio. The band intensity was normalized to the corresponding β-actin value. Cas8, caspase8; cl-Cas8, cleaved-caspase8; cl-PARP, cleaved-PARP.
